# Effects of L-carnitine against oxidative stress in human hepatocytes: involvement of peroxisome proliferator-activated receptor alpha

**DOI:** 10.1186/1423-0127-19-32

**Published:** 2012-03-21

**Authors:** Jin-Lian Li, Qiao-Yun Wang, Hai-Yun Luan, Ze-Chun Kang, Chun-Bo Wang

**Affiliations:** 1 Laboratory of Functional Physiology, Binzhou Medical University, Guanhai Road, Yantai, China; 2 Department of Pharmacology, Binzhou Medical University, Guanhai Road, Yantai, China; 3 Medical College, Qingdao University, Ningxia Road, Qingdao, China

**Keywords:** L-carnitine, Hydrogen peroxide, HL7702 cells, Antioxidant effect, Peroxisome proliferator-activated receptor alpha

## Abstract

**Background:**

Excessive oxidative stress and lipid peroxidation have been demonstrated to play important roles in the production of liver damage. L-carnitine is a natural substance and acts as a carrier for fatty acids across the inner mitochondrial membrane for subsequent beta-oxidation. It is also an antioxidant that reduces metabolic stress in the cells. Recent years L-carnitine has been proposed for treatment of various kinds of disease, including liver injury. This study was conducted to evaluate the protective effect of L-carnitine against hydrogen peroxide (H_2_O_2_)-induced cytotoxicity in a normal human hepatocyte cell line, HL7702.

**Methods:**

We analyzed cytotoxicity using MTT assay and lactate dehydrogenase (LDH) release. Antioxidant activity and lipid peroxidation were estimated by reactive oxygen species (ROS) levels, activities and protein expressions of superoxide dismutase (SOD) and catalase (CAT), and malondialdehyde (MDA) formation. Expressions of peroxisome proliferator-activated receptor (PPAR)-alpha and its target genes were evaluated by RT-PCR or western blotting. The role of PPAR-alpha in L-carnitine-enhanced expression of SOD and CAT was also explored. Statistical analysis was performed by a one-way analysis of variance, and its significance was assessed by Dennett's post-hoc test.

**Results:**

The results showed that L-carnitine protected HL7702 cells against cytotoxity induced by H_2_O_2_. This protection was related to the scavenging of ROS, the promotion of SOD and CAT activity and expression, and the prevention of lipid peroxidation in cultured HL7702 cells. The decreased expressions of PPAR-alpha, carnitine palmitoyl transferase 1 (CPT1) and acyl-CoA oxidase (ACOX) induced by H_2_O_2 _can be attenuated by L-carnitine. Besides, we also found that the promotion of SOD and CAT protein expression induced by L-carnitine was blocked by PPAR-alpha inhibitor MK886.

**Conclusions:**

Taken together, our findings suggest that L-carnitine could protect HL7702 cells against oxidative stress through the antioxidative effect and the regulation of PPAR-alpha also play an important part in the protective effect.

## Background

L-carnitine (L-3-hydroxy-4-N-N-N-trimethylaminobutyrate) is an essential nutrient that the body uses to convert fat into energy. It acts as a carrier for fatty acids across the inner mitochondrial membrane for subsequent β-oxidation [1]. It is also an antioxidant that reduces metabolic stress in the cells. Studies have reported that L-carnitine have an effective 1,1-diphenyl-2-picryl-hydrazyl (DPPH) free radical scavenging, superoxide anion radical scavenging, hydrogen peroxide scavenging, and total reducing power [2].

Recent years L-carnitine has been proposed for treatment of various kinds of disease, including liver injury. Several studies have shown that L-carnitine administration can ameliorate or prevent liver damage of various etiologies. Animal studies showed that dietary supplementation with L-carnitine could prevent hepatitis and subsequent hepatocellular carcinoma in Long-Evans Cinnamon rats [3] and alleviate alcohol-induced liver damage in rats [4]. In addition, some experimental and clinical data suggested that early intravenous supplementation with L-carnitine could improve survival in severe valproic acid -induced hepatotoxicity [5]. In vitro, L-carnitine has been successfully used to delay the killing of cultured rat hepatocytes by 1-Methyl-4-phenyl-1,2,3,6-tetrahydropyridine (MPTP) [6].

Reactive oxygen species (ROS) are considered to be involved in liver damage induced by several conditions such as alcohol abuse, fibrosis/cirrhosis of various etiologies, hepatocellular carcinoma (HCC), ischemia/reperfusion (I/R) liver injury, paracetamol overdose, and viral hepatitis [7]. Therefore, prevention or impairment of oxidative stress constitutes a therapeutic target to be achieved for hepatoprotection. Different antioxidant strategies have shown to be useful to reduce oxidative stress and cell death in hepatocytes [8]. Recently, Dobrzyńska et al. found that L-carnitine protected liver cell membranes against oxidative modifications in ethanol-intoxicated rats through its ability to scavenge free radicals [9]. Therefore, antioxidant activity of L-carnitine may make it play a role in the treatment of liver diseases.

Peroxisome proliferator-activated receptors (PPARs) are ligand-activated transcription factors belonging to the nuclear hormone receptor superfamily and are involved in energy homeostasis [10]. It consists of three members: PPAR-α, PPAR-γ, and PPAR-β/δ. PPAR-α is distributed in metabolically active tissues including liver, most prominently in hepatocytes [10,11]. PPAR-α has a central role in fatty acid oxidation, lipid and lipoprotein metabolism, inflammatory responses, and oxidative stress [12]. It was reported that PPAR-α^/^mice fed ethanol developed marked hepatomegaly, steatohepatitis, liver cell death and proliferation, and portal fibrosis [13]. PPAR-α ligands, such as Wy-14,643, were reported to have an antifibrotic action in the rat thioacetamide (TAA) model of liver cirrhosis [14]. In addition, L-carnitine treatment has been found to be able to elevate PPAR-α activation in renal tubular cells and plays a crucial role in L-carnitine anti-apoptosis effect [15]. Therefore, we hypothesize that PPAR-α may mediated the hepatoprotective effect of L-carnitine.

Our work undertaken was to determine whether L-carnitine exerts cytoprotective properties against ROS-induced cell death in cultured human hepatocytes and explores the mechanisms. For this purpose, normal human hepatocyte HL7702 was treated by H_2_O_2_, which was the major component of ROS and has been extensively used as an inducer of oxidative stress models.

## Methods

### Reagents and drugs

L-carnitine, H_2_O_2_, MTT, 2',7'-dichlorfluorescein-diacetate (DCFH-DA) and MK886 were purchased from Sigma-Aldrich (St Louis, MO, USA). Superoxide dismutase (SOD), catalase (CAT), and malonaldehyde (MDA) assay kits were all purchased from Nanjing Jiancheng Bioengineering Institute (Nanjing, China). Antibodies against PPAR-α, SOD1, and CAT were purchased from Santa Cruz Biotechnology (Santa Cruz, CA, USA). Anti-β-actin antibody was purchased from Boisynthesis Biotechnology (Beijing China). All the other reagents used were of analytical grade.

### Cells culture and treatment

Human normal hepatocyte cell line HL7702 used in this study was purchased from the Shanghai Institute of Cell Biology, Chinese Academy of Sciences (Shanghai, China). Cells were cultured in RPMI 1,640 medium supplemented with 10% fetal calf serum, 100 U/ml penicillin, and 100 μg/ml streptomycin, and maintained at 37°C in 5% CO_2 _incubator.

To estimate the effects of L-carnitine, HL7702 cells were pretreated with various concentrations of L-carnitine for 12 h before exposed to H_2_O_2_. Then, the medium was replaced with serum-free medium and 300 μM H_2_O_2 _was added. Control cells were received serum-free medium without H_2_O_2_. The cells were incubated in this condition for another 12 h.

### Cell viability analysis

Cell viability was analyzed by MTT assay. Briefly, HL7702 cells were cultured in 96-well plates at a density of 1 × 10^4 ^cells/well for 24 h. L-carnitine at different concentrations (0.01 ~ 5 mM) was added and preincubated for 12 h before exposed to H_2_O_2_. After H_2_O_2 _treatment as described, the medium was removed and cells were washed twice with PBS. Then, MTT (0.5 mg/ml) was added to each well and incubation for 4 h at 37°C. Finally, medium was removed and 150 μl dimethyl sulfoxide (DMSO) was added to each well. The plate was read using a microplate reader (Thermo Scientific, Waltham, MA, USA) at a wavelength of 570 nm.

### Measurement of lactate dehydrogenase (LDH) release

HL7702 cells were first plated in 24-well plates for 24 h. L-carnitine (0.1, 0.5, and 1 mM) was then added to the wells and preincubated for 12 h before exposed to H_2_O_2_. After H_2_O_2 _treatment, aliquots of culture medium were taken out for extracellular LDH activity analysis. Intracellular LDH activity was determined after lysis by addition of 1% Triton X-100. LDH activity was detected by automatic biochemical analyzer (Mindray, BS-200) with its commercial bio-kit at 340 nm according to manufacturer's protocol. LDH release represents the percentage of LDH in the culture medium relative to the total LDH.

### Intracellular reactive oxygen species (ROS) measurement

The fluorescent probe DCFH-DA was used to detect the intracellular ROS. DCFH-DA diffuses into cells and is hydrolyzed to form non-fluorescent 2',7'-dichlorodihydrofluorescein (DCFH). DCFH is then oxidised by ROS to form the highly fluorescent 2',7'-dichlorofluorescein. Following H_2_O_2 _treatment, cells were collected by centrifugation, resuspended in serum-free medium containing 10 μM DCFH-DA, incubated for 30 min at 37°C, washed with PBS to remove unreacted dye, resuspended in PBS and was detected at 485 nm excitation and at 535 nm emission using flow cytometry.

### Measurement of superoxide dismutase (SOD), catalase (CAT) activity and malondialdehyde (MDA) level

After H_2_O_2 _treatment, cells were resuspended in 50 mM Tris buffer containing 0.5% Triton X-100, pH 8.0, and lysed by freeze-thawing. Lysates were centrifuged at 5,000 × g for 10 min at 4°C and the supernatant obtained was used for the following measurements. The total SOD, CAT activities and MDA levels were determined spectrophotometrically using assay kits according to manufacturer's instructions. The protein content of the supernatant was determined using the Bradford method.

### Western blot analysis

PPAR-α, SOD1, and CAT protein expressions in HL7702 cells were analyzed by Western blot. Total cellular protein was extracted in lysis buffer and protein levels were quantified using a BCA (bicinchoninic acid) Protein Assay kit (Beyotime Biotechnology, Haimen, China). Aliquots containing 30 μg proteins were loaded on to a 12% SDS-polyacrylamide gel. After electrophoresis, proteins were transferred to a nitrocellulose membrane. The membrane was probed with antibodies against PPAR-α, SOD1, CAT or β-actin. The membrane was then processed with HRP conjugated goat anti-rabbit IgG (Boisynthesis Biotechnology, Beijing, China). Protein bands were visualized using the diamino-benzidine detection kit. The densities of sample bands were analyzed with Quantity One analysis software.

### Reverse transcriptase-PCR

After treatment, total RNA was extracted from HL7702 cells using Trizol reagent (Biomed, Beijing, China), according to the manufacturer's protocol. Reverse transcription to cDNA was carried out with All-in-One™ First-Strand cDNA Synthesis Kit (GeneCopoeia, USA) with oligo-dT primer in a final volume of 25 μl at 42°C for 1 h. PCR was performed using PCR Master Mix kit (Fermentas, USA) in a final volume of 50 μl. The primers used were as follows: ACT CAA CAG TTT GTG GCA AGA CA and GGA AGC ACG TCC TCA CAT GA for PPAR-α (119 bp) [16]; GGA GAG GAG ACA GAC ACC ATC CA and CAA AAT AGG CCT GAC GAC ACC TG for CPT1 (244 bp) [17]; TGT CCT ATT TGA ACG ACC TGC CCA and AGG TTC CAA GCT ACC TCC TTG CTT for ACOX (199 bp) [18]; CGT GGA AGG ACT CAT GAC CA and TCC AGG GGT CTT ACT CCT TG for GAPDH (509 bp). Specific PCR products were visualized by agarose electrophoresis. Assessment of the amount of target gene mRNA expression was performed comparatively using GAPDH mRNA as a control.

### Statistical analysis

Statistical analysis was performed by a one-way analysis of variance (ANOVA), and its significance was assessed by Dennett's post-hoc test. Data are presented as mean ± standard deviation (SD), and a value of *P *< 0.05 was considered statistically significant.

## Results

### L-carnitine protected HL7702 cells against H_2_O_2_-induced cytotoxicity

**Figure 1:**
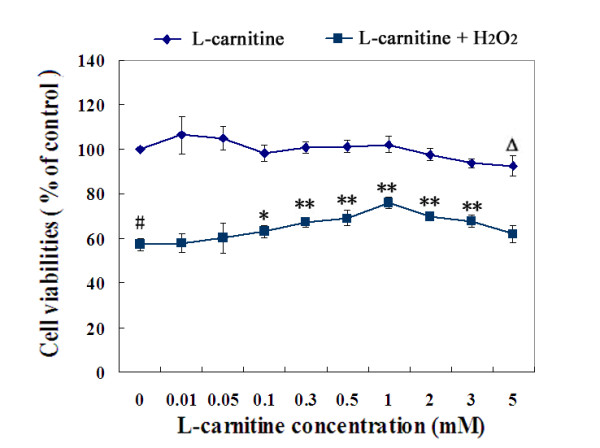
**The cytotoxicity of L-carnitine and its protective effect on H_2_O_2_-induced toxicity in HL7702 Cells**. The upper curve: HL7702 cells were incubated with L-carnitine for 24 h. The lower curve: Cells were treated with or without L-carnitine prior to H_2_O_2 _challenge (300 μM, 12 h). Cell viability was assayed by MTT assay. The percentage of cell viability in the control group was treated as 100%. Each value represents the mean of three replicates. ^Δ^*P *< 0.05, ^#^*P *< 0.01 *vs*. control; *P < 0.05, **P < 0.01 *vs*. H_2_O_2 _alone.

Cell viability was evaluated by MTT assay. To determine the cytotoxicity of L-carnitine and what concentrations of L-carnitine may exert a cytoprotective effect on H_2_O_2_-induced toxicity in HL7702 cells, dose-viability curves were generated, as shown in Figure 1.

The upper curve in Figure 1 showed that, coincubated with L-carnitine, concentrations ranging from 0.01 to 3 mM, cell growth were not be affected significantly, while at a higher concentration of 5 mM, a significant inhibitory effect of L-carnitine was observed (*P *< 0.05). This finding suggests that the toxicity of L-carnitine increases after 3 mM.

The lower curve in Figure 1 showed that, after exposure to 300 μM H_2_O_2 _for 12 h, HL7702 cells displayed markedly decreased viability compared to untreated ones. Doses of L-carnitine ranging from 0.1 to 3 mM significantly reduced the cell viability loss, and a dose-dependent manner was found from 0.1 to 1 mM, we thus decided to use 0.1 ~ 1 mM for all subsequent experiments.

### L-carnitine inhibited H_2_O_2_-induced LDH release in HL7702 cells

**Figure 2:**
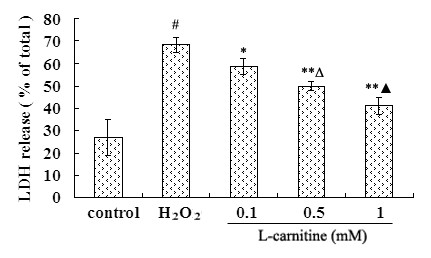
**Effect of L-carnitine on the LDH release of HL7702 cells**. Cells were treated with or without L-carnitine prior to H_2_O_2 _challenge (300 μM, 12 h). LDH release represents the percentage of LDH in the culture medium relative to the total LDH. Each value represents the mean of three replicates. ^#^*P *< 0.01 *vs*. control; *P < 0.05, **P < 0.01 *vs*. H_2_O_2 _alone; ^Δ^*P *< 0.05 *vs*. 0.1 mM L-carnitine; ^▲^*P *< 0.05 *vs*. 0.5 mM L-carnitine.

The integrity of cell membrane was determined by the release of LDH. Figure 2 showed that there was a low LDH leakage ratio under normal conditions. However, H_2_O_2 _induced significant LDH release. Pretreatment with 0.1 ~ 1 mM L-carnitine reduced H_2_O_2_-induced LDH release in a dose-dependent manner.

### L-carnitine promoted endogenous antioxidant defense in HL7702 cells under oxidative stress

**Figure 3:**
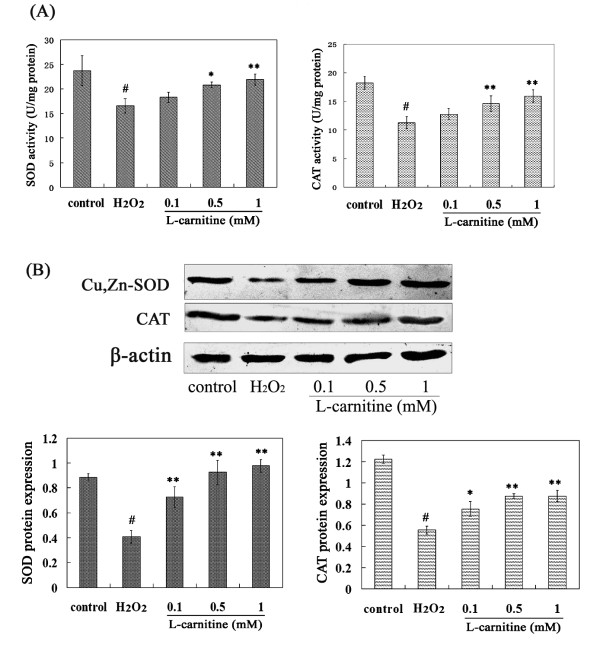
**Effect of L-carnitine on the activities and protein expressions of SOD and CAT in H_2_O_2_-damaged HL7702 cells**. Cells were treated with L-carnitine for 12 h and followed by the treatment of H_2_O_2 _(300 μM) for 12 h. A: Total SOD and CAT activities were calculated in units of activity per mg of total protein. B: Cu,Zn-SOD and CAT expressions were determined by western blot analysis. Results were expressed as the ratio of expression level of Cu,Zn-SOD or CAT over β-actin. Each value represents the mean of three replicates. ^#^*P *< 0.01 *vs*. control; *P < 0.05, **P < 0.01 *vs*. H_2_O_2 _alone.

SOD, CAT activities and protein levels were measured to investigate the antioxidative effect of L-carnitine on H_2_O_2_-damaged HL7702 cells. The activity was expressed as U/mg protein in cells. Compared with the control group, the activities of total SOD and CAT in the H_2_O_2 _alone group were decreased by 30.05% and 38.25% respectively (Figure 3A). Compared to H_2_O_2_-damaged cells, 0.5 ~ 1 mM L-carnitine reversed the activities of SOD and CAT. Western blot analysis showed that H_2_O_2 _exposure resulted in a marked decrease of Cu,Zn-SOD and CAT protein levels in HL7702 cells, while L-carnitine pretreatment significantly elevated the protein levels (Figure 3B).

### L-carnitine scavenged H_2_O_2_-induced ROS production in HL7702 cells

**Figure 4:**
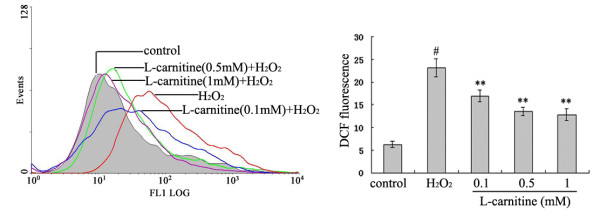
**Effect of L-carnitine on intracellular ROS levels after H_2_O_2 _exposure in HL7702 cells**. Cells were treated with or without L-carnitine prior to H_2_O_2 _challenge (300 μM, 12 h). ROS levels were measured using fluorescent probe DCFH-DA. Each value represents the mean of three replicates. ^#^*P *< 0.01 *vs*. control; **P < 0.01 *vs*. H_2_O_2 _alone.

To determine whether L-carnitine prevents H_2_O_2_-induced ROS generation, the concentration of intracellular ROS was evaluated by the changes in DCF fluorescence intensity. The result showed that DCF fluorescence intensity dropped significantly from 23.1 ± 2.0 in cells treated with H_2_O_2 _only to values between 16.9 ± 1.3 and 12.8 ± 1.3 in cells to which 0.1 ~ 1 mM L-carnitine was added (Figure 4).

### L-carnitine inhibited lipid peroxidation in H_2_O_2_-damaged HL7702 cells

**Figure 5:**
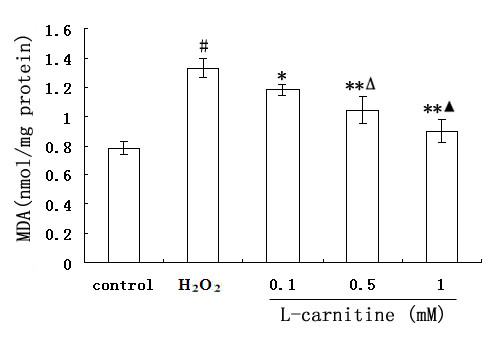
**Effect of L-carnitine on H_2_O_2_-induced MDA formation in HL7702 cells**. Cells were treated with or without L-carnitine prior to H_2_O_2 _challenge (300 μM, 12 h). Each value represents the mean of three replicates. ^#^*P *< 0.01 *vs*. control; *P < 0.05, **P < 0.01 *vs*. H_2_O_2 _alone; ^Δ^*P *< 0.05 *vs*. 0.1 mM L-carnitine; ^▲^*P *< 0.05 *vs*. 0.5 mM L-carnitine.

The ability of L-carnitine to inhibit lipid peroxidation in H_2_O_2_-treated HL7702 cells was also tested by determining MDA levels. As shown in Figure 5, the exposure of cells to H_2_O_2 _increased MDA levels by approximately 1.7 fold relative to control cells. An obvious dose-dependent reduction of L-carnitine on MDA levels in H_2_O_2_-treated cells was observed.

### L-carnitine up-regulated PPAR-α expression in H_2_O_2_-damaged HL7702 cells

**Figure 6:**
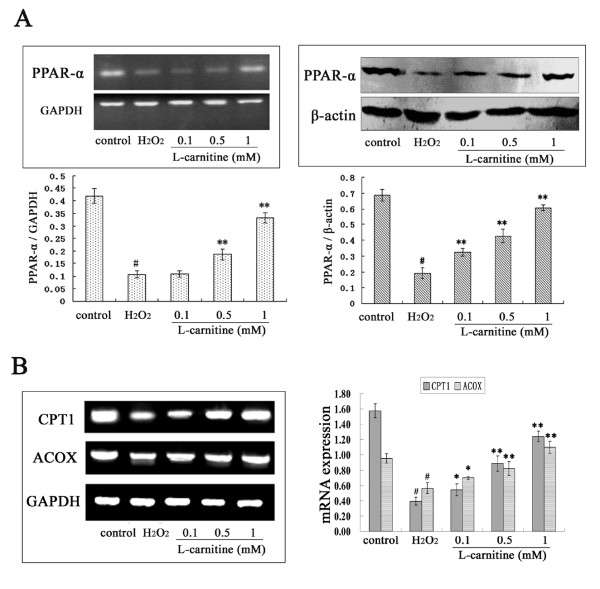
**Effect of L-carnitine on expression levels of PPAR-α and its target genes in H_2_O_2_-treated HL7702 cells**. HL7702 cells were pretreated with L-carnitine for 12 h and followed by the treatment of H_2_O_2 _(300 μM) for 12 h. A: Cells were collected, total RNA and protein were prepared to determine the mRNA and protein levels of PPAR-α in HL7702 cells using RT-PCR and western blot analysis respectively. B: Total RNA was extracted and expressions of CPT1 and ACOX were quantified using RT-PCR. The data was normalized to GAPDH expression. Each value represents the mean of three replicates. ^#^*P *< 0.01 *vs*. control; *P < 0.05, **P < 0.01 *vs*. H_2_O_2 _alone.

We further analyzed the effect of L-carnitine on PPAR-α mRNA and protein expressions in H_2_O_2_-treated HL7702 cells. As shown in Figure 6, treatment of HL7702 cells with 300 μM H_2_O_2 _for 12 h reduced the PPAR-α mRNA and protein levels in HL7702 cells. When the cells were preincubated with L-carnitine before H_2_O_2 _exposure, PPAR-α expression increased markedly compared to H_2_O_2 _alone group, although the mRNA expression was not significantly changed in 0.1 mM L-carnitine group.

To assess if the elevated PPAR-α expression by L-carnitine leads to induction of PPAR-α-regulated genes in H_2_O_2_-treated HL7702 cells, we examined mRNA levels of CPT1 and ACOX by RT-PCR. Exposure to H_2_O_2 _caused inhibition of the mRNA expression of CPT1 and ACOX. L-carnitine, on the other hand, attenuated the inhibitory effect of H_2_O_2_.

### L-carnitine increases SOD and CAT expression in H_2_O_2_-damaged HL7702 cells via PPAR-α expression

**Figure 7:**
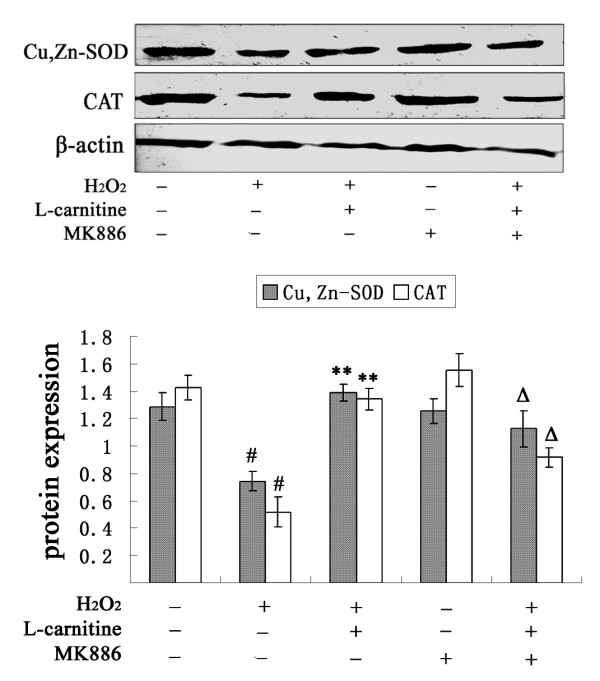
**Influence of MK886 on SOD and CAT protein expression enhanced by L-carnitine in H_2_O_2_-treated HL7702 cells**. HL7702 cells were pretreated with L-carnitine (1 mM) for 12 h prior to H_2_O_2 _challenge (300 μM, 12 h). MK886 (5 μM) was added to cells 2 h prior to H_2_O_2 _insult. Cu,Zn-SOD and CAT expressions were determined by western blot analysis. ^#^*P *< 0.01 *vs*. control; **P < 0.01 *vs*. H_2_O_2 _alone; ^Δ^*P *< 0.05 *vs*. L-carnitine group. The presented results are representative of three independent experiments.

Activation of PPAR-α by agonists has been previously shown to enhance SOD expression and CAT activity in the liver [19,20]. In order to investigate whether the elevated SOD and CAT expression induced by L-carnitine was due to enhanced levels of PPAR-α, a specific antagonist of PPAR-α, MK886, was used. MK886 (5 μM) was added to cells 2 h prior to H_2_O_2 _insult. As shown in Figure 7, MK886 did not alter SOD and CAT protein levels in control cells. However, the up-regulation of SOD and CAT expression by L-carnitine was inhibited by addition of MK886 in H_2_O_2_-treated cells.

## Discussion

H_2_O_2 _is a major component of ROS produced intracellularly during many physiological and pathological processes, and causes oxidative damage. It has been extensively used as an inducer of oxidative stress in vitro models. Our results showed that 300 μM H_2_O_2 _exposure for 12 h induced a significantly decreased cell growth and elevated LDH leakage in HL7702 cells. L-carnitine at a concentration of 0.1 to 3 mM showed no harmful effects on the growth of HL7702 cells, however, cell growth was inhibited by 5 mM L-carnitine. The possibility may be that high doses of carnitine possess pro-oxidant activity as has been reported [21]. Our results demonstrated the protective effects of 0.1 ~ 3 mM L-carnitine against the inhibition of cell growth induced by H_2_O_2_. LDH leakage was also inhibited by 0.1 ~ 1 mM L-carnitine in a dose-dependent manner. These findings suggest that L-carnitine is capable of reducing H_2_O_2_-induced cytotoxicity in HL7702 cells.

Oxidative stress after H_2_O_2 _exposure results from intracellular ROS production and decreased ROS scavenging. To further explore the mechanism of the protective effect of L-carnitine on H_2_O_2_-induced oxidative damage in HL7702 cells, we examined the effect of L-carnitine on intracellular ROS production. H_2_O_2 _challenge caused an apparent increase in ROS levels in HL7702 cells compared to control group, however, in vitro treatment with L-carnitine resulted in reduced ROS levels. Scavenging of ROS is determined by antioxidant enzymes such as SOD and CAT. CAT is considered to be the most relevant enzyme involved in detoxification of H_2_O_2 _and protection of hepatocytes from oxidative stress [22]. We observed that the inhibited activities and expressions of SOD and CAT induced by H_2_O_2 _were attenuated by L-carnitine. These observations supported the idea that L-carnitine did protect HL7702 cells from H_2_O_2_-induced cytotoxicity by its antioxidant property.

Liver damage was associated with enhanced lipid peroxidation and formation of lipid radicals [23,24]. MDA, as an end product of lipid peroxidation, usually used to estimate the extent of lipid peroxidation. It has been shown that many pathological conditions that resulted in elevation of MDA due to lipid peroxidation were prevented by L-carnitine [25,26]. In our paper, MDA levels in HL7702 cells increased after H_2_O_2 _exposure. However, pretreatment with L-carnitine (0.1 ~ 1 mM) prevented a further increase in MDA levels.

H_2_O_2_, a low molecular weight compound, can easily penetrate lipid membrane, cause lipid peroxidation, and disturb lipid homeostasis. PPAR-α is a ligand-dependent transcription factor that is known to have critical roles in the regulation of lipid homeostasis. It has been reported that PPAR-α expression decreased in a rat model of non-alcoholic fatty liver disease [27] and the absence of PPAR-α have been demonstrated to cause lipid accumulation in liver of rats [28]. Our results showed that HL7702 cells exposed to 300 μM H_2_O_2 _for 12 h showed a significant reduction in PPAR-α expression levels, indicating the disturbed lipid homeostasis might occur in H_2_O_2_-damaged HL7702 cells. However, L-carnitine pretreatment attenuated the inhibitory effect of H_2_O_2 _on the expression of PPAR-α. It is well known that expression of a range of genes involved in lipid homeostasis is controlled by PPAR-α, which binds to the peroxisome proliferator response element (PPRE) in the promoters of these genes [29]. Therefore, mRNA expression of CPT1 and ACOX, two PPAR-α target genes that control fatty acid oxidation [30,31], were also investigated. Results showed that the repression of CPT1 and ACOX expression induced by H_2_O_2 _were all attenuated by L-carnitine. Above observations indicate that the disturbed lipid homeostasis induced by H_2_O_2 _might be ameliorated by L-carnitine by increasing PPAR-α expression. In fact, as we know, L-carnitine acts as a carrier participate in fatty acids β-oxidation. So, the attenuated lipid metabolism in H_2_O_2_-damaged HL7702 cells would be significantly ameliorated by L-carnitine. This observation is consistent with a recent study, which demonstrated that L-carnitine supplementation induced recovery of liver lipid metabolism in cachectic animals [32]. Furthermore, it is likely that the enhancement of β-oxidation induced by L-carnitine would generate ATP, thereby reversing H_2_O_2_-initiated depletion of ATP in cells and attenuating cell injury. ATP was considered to be a critical event in lethal cell injury produced by oxygen radicals [33]. The hypotheses need further investigation.

It has been reported that PPAR-α gene expression is associated with SOD gene expression in the liver [20], and CAT has been identified as one of the target enzymes of PPAR-α [34]. In the present study, the up-regulation of SOD and CAT expression by L-carnitine were attenuated by PPAR-α antagonist MK886. The results reveal the crucial role of PPAR-α activation in the protective effect of L-carnitine against H_2_O_2_-induced damage in HL7702 cells. L-carnitine might elevate PPAR-α expression, and then activate SOD and CAT, resulting in a decrease in extracellular H_2_O_2 _levels and prevention of liver damage.

## Conclusions

Taken together, the present results provide evidence that L-carnitine prevented in vitro human hepatocyte oxidative stress induced by H_2_O_2_. The protective effects of L-carnitine observed in the current paper can possibly be mediated through its antioxidant potential. The elevated PPAR-α expression by L-carnitine play an important part in the protective effect, which might contribute to the amelioration of lipid homeostasis, the improvement of antioxidant ability, and increased ATP in L-carnitine treated cells.

## Abbreviations

H_2_O_2_: Hydrogen peroxide; MTT: 3-[4,5-dimethylthiazol-2-yl]-2,5-diphenyltetrazolium bromide; LDH: Lactate dehydrogenase; ROS: Reactive oxygen species; SOD: Superoxide dismutase; CAT: Catalase; MDA: Malondialdehyde; PPAR: Peroxisome proliferator-activated receptor; CPT1: Carnitine palmitoyl transferase 1; ACOX: Acyl-CoA oxidase; DPPH: 1,1-diphenyl-2-picryl-hydrazyl; DCFH-DA: 2,7-dichlorofluorescein diacetate; DMSO: Dimethyl sulfoxide.

## Competing interests

The authors declare that they have no competing interests.

## Authors' contributions

JLL designed, carried out the experiment and drafted the manuscript. QYW and HYL participated in the design and coordination of this study, and performed experiments. ZCK performed the partial experiments and analyzed data. CBW participated in the analysis and interpretation of data. All authors read and approved the final manuscript.
